# Malignant Transformation of Mature Cystic Teratoma Diagnosed after a 10-Year Interval

**DOI:** 10.1155/2017/2947927

**Published:** 2017-05-21

**Authors:** Mariko Jitsumori, Satoru Munakata, Toshiya Yamamoto

**Affiliations:** ^1^Department of Obstetrics and Gynecology, Sakai City Medical Center, Sakai, Japan; ^2^Department of Pathology, Sakai City Medical Center, Sakai, Japan

## Abstract

A 67-year-old uniparous woman had undergone surgery for acute perforated gastric ulcer 10 years prior to the current presentation. Although abdominal computed tomography (CT) performed at that point had revealed a mature cystic teratoma measuring 6 cm in diameter in the right ovary, it was left untreated. She visited the department of surgery at our hospital with chief complaints of appetite loss, nausea, and vomiting that had persisted for the prior two weeks. She was admitted with a diagnosis of ileus attributed to an abdominal incisional hernia. CT performed on admission revealed a tumor measuring 21 cm in diameter with mural nodules in the right ovary. Thus, surgery was performed under suspicion of malignant transformation of the previously detected ovarian mature cystic teratoma. While neither lymphadenopathy nor distant metastasis was detected by imaging studies, bilateral adnexectomy and repair of the abdominal incisional hernia were performed. Cytology of ascites was negative. The postoperative pathological diagnosis was squamous cell carcinoma arising from teratoma, and the postoperative clinical diagnosis was stage IA ovarian cancer. It was assumed that the mature cystic teratoma which had been detected in the right ovary 10 years earlier had undergone malignant transformation.

## 1. Introduction

Mature cystic teratoma, which is the most common benign ovarian tumor type, is known to occasionally undergo malignant transformation. Malignant transformation of mature cystic teratoma is extremely difficult either to predict or to detect early. Moreover, the mechanism of malignant transformation has not as yet been elucidated. We experienced a case with a mature cystic teratoma that had undergone malignant transformation over a period of 10 years. We herein present this case with a literature review.

## 2. Case

**Figure 1:**
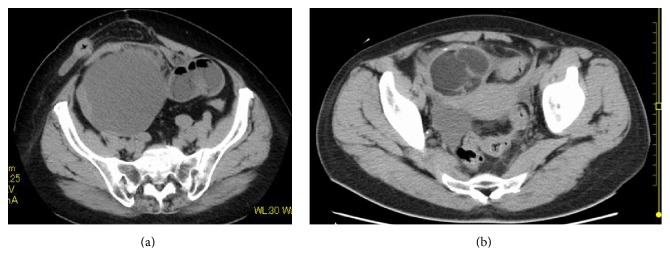
(a) Abdominal plain computed tomography (current). A huge mass measuring 21 cm in longest diameter can be seen in the right cranial portion of the uterine body. The mass is partially solid, suggesting malignancy. (b) Abdominal plain computed tomography (image obtained 10 years prior to the current admission). A mature cystic teratoma measuring 6 cm in diameter is observed on the right side. There are no findings suggestive of malignancy, such as a mural nodule or a solid component.

**Figure 2:**
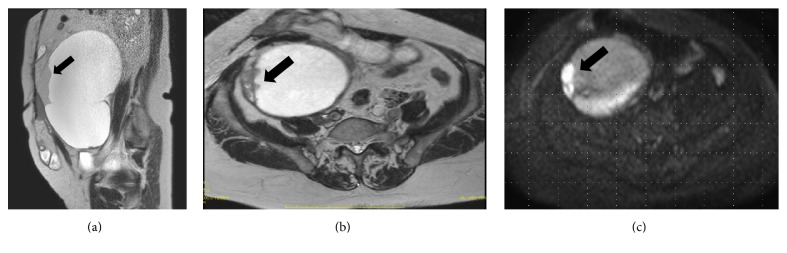
Pelvic plain magnetic resonance imaging. (a) T2-weighted image in the sagittal plane. A cyst measuring 21 × 19 × 12 cm is observed. It is partially solid. (b) T2-weighted image in the horizontal plane. (c) Diffusion-weighted image in the horizontal plane. The solid components observed on the T2-weighted image show high signal intensity on the diffusion-weighted image (*⬅*). The cyst is suspected to be a malignant lesion.

**Figure 3:**
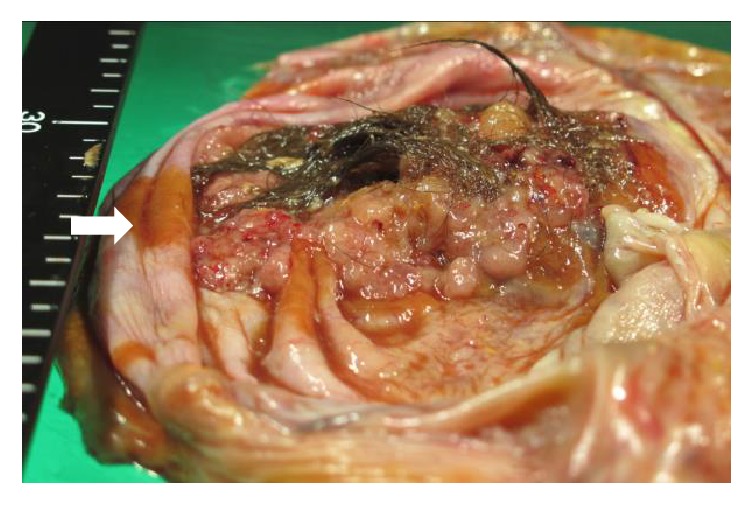
Resected specimen. Protruding lesions are observed on a portion of the wall of the right ovarian tumor.

**Figure 4:**
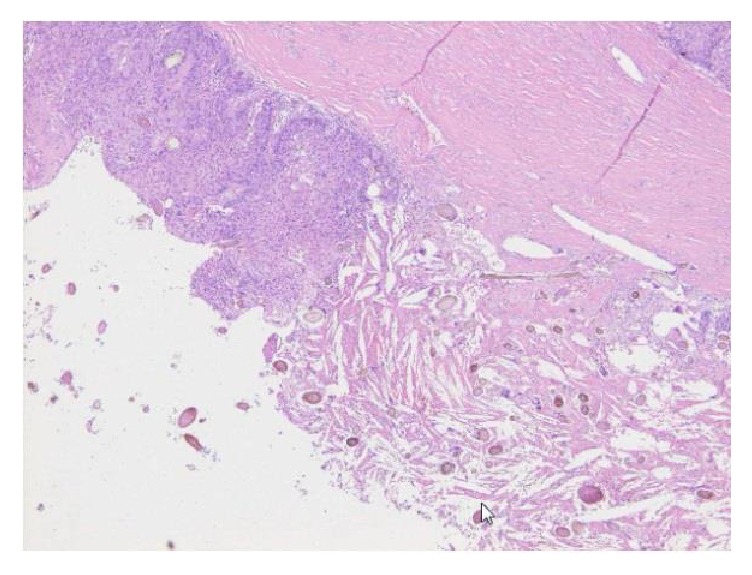
Histologically, squamous cell carcinoma was observed inside the cyst (left side of the picture). Granulation tissue containing hair was also found (right side of the picture) (H&E staining, ×100).

Our patient was a 67-year-old woman, gravida 4, para 1. Her past medical history included epilepsy diagnosed at 56 years of age, unspecified cardiopulmonary arrest at age 57 years, peritonitis due to acute perforated gastric ulcer, venous thrombosis of the lower limb, and pulmonary arterial embolism. She was also allergic to numerous drugs and diagnostic agents (e.g., contrast media, nonsteroidal anti-inflammatory drugs, and sodium valproate). She visited the department of surgery at our hospital with chief complaints of appetite loss, nausea, and vomiting that had persisted for the prior two weeks. Because abdominal plain computed tomography (CT) revealed ileus and an abdominal incisional hernia, she was immediately admitted with a diagnosis of ileus caused by the incisional hernia. Moreover, a tumor measuring 21 cm in longest diameter was detected in the pelvis (Figure 1(a)). She was thus referred to our department for detailed examination and treatment. At the initial examination, the abdomen was soft without tenderness, rebound tenderness, or muscular defense. An easily movable mass extending from the right lower abdomen to the level of the umbilicus was palpated. The patient had undergone omental implantation for acute perforated gastric ulcer 10 years earlier. Preoperative abdominal plain CT had revealed a right ovarian tumor measuring 6 cm in diameter (Figure 1(b)), which contained a part of calcification and fatty components; however, there had been no findings suggestive of malignancy, such as a solid component or a mural nodule. The right ovarian tumor was radiologically diagnosed with a mature cystic teratoma. After surgery for the acute perforated gastric ulcer, she had not been referred to the department of gynecology. No further examination of the right ovarian tumor was performed. She had not been followed up for the ovarian tumor. When the findings of abdominal plain CT performed during the current admission were compared to those of the abdominal CT obtained 10 years earlier, the ovarian tumor was noted to have grown markedly in size and to be partially solid. The CT performed during the current admission also revealed fatty components in the ovarian cyst. On the basis of these findings, malignant transformation of the mature cystic teratoma was suspected. Furthermore, pelvic plain magnetic resonance imaging (MRI) also showed a cyst measuring 21 cm in longest diameter that was partially solid on the right side of the uterine body (Figure 2(a)). The solid components detected on T2-weighted images (Figure 2(b)) showed high signal intensity on diffusion-weighted images (Figure 2(c)) and low signal intensity on apparent diffusion coefficient maps, which suggested a malignant lesion. In addition, blood tests revealed tumor marker elevations: CEA, 7.1 ng/mL (<4.9 ng/mL); CA125, 58.3 U/mL (<35 U/mL); CA19-9, 405.8 U/mL (<37 U/mL); and SCC antigen, 6.2 ng/mL (<1.5 ng/mL). Based on the clinical course, imaging findings, and elevated tumor markers, malignant transformation of the previously recognized mature cystic teratoma was strongly suspected. Sixteen days after the initial examination, semiurgent surgery was performed. While neither lymphadenopathy nor distant metastasis was detected by imaging studies, the operation consisted of abdominal bilateral adnexectomy and repair of the abdominal incisional hernia in consideration of the patient's general condition. The intraoperative findings included slight accumulation of ascites with a pink tinge due to blood and swelling of the right ovary, which was larger than a newborn's head, whereas there were no signs of capsule rupture, torsion abnormality, and so on. We detected no macroscopic abnormalities in the uterus or the left adnexa. Neither disseminated lesions nor lymphadenopathy was observed in the peritoneal cavity. The tumor in the right ovary was unilocular and weighed 2960 g, containing both fatty components and hair. Moreover, protruding lesions were observed on a portion of the tumor wall (Figure 3). Cytology of ascites was negative. Histologically, cystic wall was lined by squamous epithelium and contained horny materials inside the cyst. Mature bone tissue and hair were also observed. Focally, granulation tissue was formed. Squamous cell carcinoma was found in the solid part protruding inside the cyst wall. There was a transition between squamous epithelium and squamous cell carcinoma (Figure 4). The postoperative clinical diagnosis was ovarian cancer FIGO stage IA, pT1aNxM0 due to malignant transformation of a mature cystic teratoma which had first been noted 10 years earlier. Given the history of allergy to multiple drugs, cardiopulmonary arrest, venous thrombosis of the lower limb, and pulmonary arterial embolism, postoperative chemotherapy was not planned. As of two years since surgery, no recurrence has been observed.

## 3. Discussion

Mature cystic teratoma, which is a commonly observed benign ovarian tumor in young women, is a germ cell tumor containing fat, hair, teeth, cartilage, and so forth. It is the most common type of benign ovarian tumor [1] accounting for approximately 20% of all ovarian tumors according to Hurwitz et al. [2]. A quarter of ovarian tumors are reportedly of the germ cell type, most of which are mature cystic teratomas [3]. Mature cystic teratoma is known to occasionally become malignant, and the majority of such transformations result in SCC. Malignant transformation was reported in 1.8% of 8000 patients with a mature cystic teratoma by Peterson [4] and 1% to 2% of such patients by Hurwitz et al. [2], while Kim et al. reported that malignant transformation was observed in 4 of 560 patients (0.6%) who underwent surgery for a mature cystic teratoma at their facility [5]. According to histological types, SCC accounts for the majority of cases, 80% to 90% according to Hurwitz et al. [2] and approximately 80% as reported by Hackethal et al. [3]. Adenocarcinoma [2, 6] and malignant melanoma [6] have also been reported. Kikkawa et al. reported that 42 cases with a mature cystic teratoma undergoing malignant transformation included 37 cases developing SCC, while the remaining cases had an adenocarcinoma or malignant melanoma [6]. According to a review of 277 cases with malignant transformation of a mature cystic teratoma into SCC described in 64 articles with sufficient data that were selected from among 126 articles published between 1978 and 2007, the mean age was 55 years, and the tumor diameter was 10 cm or longer in many cases [3, 7]. Moreover, Hackethal et al. reported that the diameter exceeded 100 mm in several cases with mature cystic teratoma undergoing malignant transformation [3]. Chen et al. reported that the cut-off value was 137 ± 57 mm [8], while Kikkawa et al. reported that it was 99 mm [9]. Regarding age, the reported mean ages were 48 [2] and 55 ± 14.4 years [8], whereas malignant transformation is reportedly common in patients 50 years of age or older [3]. As for diagnostic criteria, the reported cut-off values for age were 40 [5, 10] and 45 years [9].

Malignant transformation of a mature cystic teratoma is mainly detected by diagnostic imaging. It is considered to be important to focus on the presence or absence of solid components on pelvic MRI images [11]. In SCC cases, the SCC antigen is regarded as being a useful tumor marker [7]. Kikkawa et al. performed screening by measuring SCC antigen (<2.0 ng/mL), CA 125 (<35 U/mL), CA 19-9 (<37 U/mL), and CEA (<5.0 ng/mL) levels in cases with malignantly transformed mature cystic teratomas, reporting that diagnostic efficiencies were 63%, 50%, 28%, and 45%, respectively [9]. In our 67-year-old patient with SCC measuring 21 cm in diameter, these tumor markers (CEA, 7.1 ng/mL; CA 125, 58.3 U/mL; CA 19-9, 405.8 U/mL; and SCC antigen, 6.2 ng/mL) were elevated, and MRI revealed solid components suggestive of malignancy. Thus, in terms of age, histological type, tumor diameter, tumor marker elevations, and MRI findings, our case represents a typical example of the generally known features of patients with malignant transformation of a mature cystic teratoma. Although mature cystic teratoma is a frequently observed benign ovarian tumor, it may become malignant as seen in our case. Thus, these teratomas require continuous follow-up, and patients also need to be informed about the possibility of malignant transformation at the time of explanation.

Although the mechanism of malignant transformation of a mature cystic teratoma remains unknown, involvement of the human papilloma virus has been suggested [12, 13]. Moreover, no reports have mentioned how long it takes for a mature cystic teratoma to become malignant. In fact, it is difficult to estimate when malignant transformation occurred in our case. However, because the cancer was diagnosed as stage IA despite an interval of 10 years, we assume that malignant transformation had progressed very slowly. The peak age at diagnosis of mature cystic teratoma and the mean age of patients experiencing malignant transformation of a teratoma differ by at least 10 years, though there is one report describing an 85-year-old patient with a mature cystic teratoma that did not undergo malignant transformation [14]. Thus, malignant transformation appears to take a very long time. However, because it is difficult to predict or achieve early detection of malignant transformation, caution must be exercised when patients with mature cystic teratomas are followed up long-term.

We experienced a case with a mature cystic teratoma which underwent malignant transformation during an interval of 10 years since its initial detection. Although mature cystic teratoma is a benign tumor, surgery or regular follow-up needs to be planned after due consideration of the risk of malignant transformation.
